# Effects of root pruning radius and time on yield of tuberous roots and resource allocation in a crop of *Helianthus tuberosus* L.

**DOI:** 10.1038/s41598-018-22586-1

**Published:** 2018-03-13

**Authors:** Kai Gao, Tiexia Zhu, Lin Wang, Yang Gao

**Affiliations:** Inner Mongolia University for Nationalities, Tongliao, Inner Mongolia 028000 China

## Abstract

The production of tuberous roots is usually reduced by vigorous vegetative growth because of the competition for resource between the vegetative parts and reproductive organs. In this study, we conducted root pruning to examine the vigorous vegetative growth by regulating root growth, subsequently limiting vegetative growth and improving tuber yield. Compared with the control, stem, tuber, and root biomasses were all improved, whereas both flower and leaf biomasses were increased. Tuber biomass was improved by 23.48% to 50.32%, with the largest tuber biomass obtained at root cutting radius 4/5 R. With delayed root cutting time, tuber and root biomasses increased first and then decreased. The largest tuber biomass was obtained at 65 seedling stage. With a delay in root cutting time, the trend line of aboveground, underground, and total biomasses changed gradually. However, whereas underground and total biomasses showed a gradually increasing, aboveground biomass showed a decreasing. The values of stem-leaf and shoot-root ratios under different root cutting were higher than those of the control. With a delay in root cutting time, stem-leaf ratio showed an initial increase and then decreased with largest value being obtained at 80 seedling stage, whereas the largest shoot-root ratio was obtained at 115 seedling stage.

## Introduction

*Helianthus tuberosus* L., commonly known as Jerusalem artichoke (JA), is coming from the temperate regions of North America, which is usually grown for its tubers^1^. The tubers of JA are rich in inulin, a fructose oligosaccharide that has been used as a raw material of sugars and bio-ethanol. Previous studies have indicated that the tubers of JA comprise approximately 70–90% inulin.The inulin is a fructose polymer that can be decomposed into a single fructose by exoinulinase. It can then be fermented to produce ethanol with a conversion rate of 83–99%^1–3^.

One of the most important elements in *H. tuberosus*. management is vegetative growth regulation. Previous studies have shown that the dry weight of Jerusalem artichoke aboveground biomass (including flowers, leaves, and stems) ranges from 6 to 9 tons per hectare in poor conditions to 20 to 30 tons per hectare in good conditions, and that the yield of tubers ranges from 2 to 3 tons per hectare to 10 to 15 tons per hectare^4^. However, for the production of bio-ethanol and inulin, the raw material is tuber sections. In this regard, there have been numerous studies on factors, such as harvest time, fertilizer management, water management, and planting density, in order to improve the yield of tubers^5–8^. Although the yield of tubers has been improved by optimum management during cultivation of the Jerusalem artichoke, it has yet to be determined whether, for example, the yield of tubers can be improved by applying nitrogen fertilizer. Furthermore, excessive stem and leaf growth have been shown to affect tuber yield^9–11^. In this regard, some studies have indicated that assimilate accumulation, transportation, and distribution all have a direct relationship with crop yield, and that they are mainly affected by source-sink relationships, which can be altered by various management measures^1,12^.

Many methods are available for regulating vegetative growth in crops and trees^13^. Root pruning is a common technique that can reduce the vegetative growth of crops and trees, which is a highly effective and economical method that not only assists in dwarfing but can also be used to stimulate the new roots necessary to sustain growth^14^. Root pruning disrupts the old growth balances of crops and trees and alters their assimilation abilities, nutrient distributions, and hormone level. However, to the best of our knowledge, there have been no previous studies that have examined the application of root pruning in *H. tuberosus*. Accordingly, it is currently not known whether root pruning can be used as an alternative method for regulation of the vegetative growth of *H. tuberosus*, and if so, how this method will influence tuber yield and the biomass of other organs. Therefore, it is desirable to determine the performances of root pruning in the production of *H. tuberosus*.

We hypothesized that root pruning would be useful in regulating the vigorous vegetative growth of *H. tuberosus*, and accordingly examined the influence of cutting time and radius on biomass and matter allocation.

## Methods

### Plant material and growth conditions

The experiment was conducted at the experimental field station of the Inner Mongolia University for the Nationalities in Tongliao (Inner Mongolia, China.). It is a semi-arid region with a temperate monsoon climate. Yearly precipitation is approximately 399 mm, and 50–60% of the annual rain falls in August and September. The mean annual temperature is 6.4 °C. The annual accumulated temperature above 10 °C is 31844 °C. The frost-free period is approximately 150 d from May to September. The soil type is grey meadow soil. The organic matter content is approximately 18 g/kg, and available nitrogen, phosphorus, and potassium are approximately 62, 39, and 185 mg/kg, respectively. The pH is 8.2.

In 2016, whole tubers within the weight range of 20 to 25 g were used in the experiments. Tubers were hand-planted in 200-cm-long and 200-cm-spaced rows, and there were 2500 plants per hectare. Planting was carried out on 1^st^ May 2016. The plot area was 20 m × 20 m, with five repeats. Nitrogen, phosphorus and potassium were applied at the rate of 80, 20, and 40 kg ha^−1^, respectively, based on the results of pre-plant soil analysis and taking into account the nutritional needs of JA.

### Experiment design

Five cutting times and five cutting radii were used according to JA root horizontal distribution characteristics. The cutting radius was confirmed by the actual root length of different grow stages, as follows:

- Treatment Time: 50 seedling stage (T1), 65 seedling stage (T2), 80 seedling stage (T3), 95 seedling stage (T4), and 115 seedling stage (T5)
- Treatment Radius: cutting radius 1/5 R (R1), cutting radius 1/4 R (R2), cutting radius 1/3 R (R3), cutting radius 1/2 R (R4), and no cutting (R5)
- The individual treatment were as follows: T1R1, T1R2, T1R3, T1R4, T1R5, T2R1, T2R2, T2R3, T2R4, T2R5, T3R1, T3R2, T3R3, T3R4, T3R5, T4R1,T4R2, T4R3, T4R4, T4R5, T5R1, T5R2, T5R3, T5R4, and T5R5.
- “R” indicates the actual length during cutting stage. The length value was obtained by testing when cutting roots.

#### Data collection and determination methods

On 14^th^ October 2016, we collected samples, including aboveground and underground biomass, and separated the roots, stems, leaves, branches, flowers, and tubers. Underground biomass was taken at a depth of 50 cm and 100 cm radius. The samples were collected from five JA plants that were randomly selected as experimental subjects in each plot. All samples were dried in an oven at 75 °C to determine dry weight and stem-leaf and shoot-root ratios were calculated.

### Calculation and Data Analysis

Analysis of variance was conducted utilizing a two-factor analysis of variance and Correlation Analysis using SPSS 17.0 (SPSS Inc., Chicago IL, USA).

## Results

### The influence of root cutting time and radius on biomass in JA

We observed a significant influence of root cutting time on stem biomass, leaf biomass, and tuber biomass (P < 0.01), and an influence on underground biomass (P < 0.05). There was a marked influences of root cutting radius on stem biomass, leaf biomass, tuber biomass, root biomass, and underground biomass (P < 0.01), and an influence on flower biomass (P < 0.05) (Table 1).

**Table 1:** Analysis of Variance of the effect of cutting time and cutting radius on organs biomass.

		Cutting Time	Cutting Radius			Cutting Time	Cutting Radius
Stem Biomass	F value	15.1155	5.2952	Root Biomass	F value	1.8043	35.1810
	P value	0.0000	0.0013		P value	0.1434	0.0000
Leaf Biomass	F value	4.6674	5.8724	Underground Biomass	F value	3.4375	56.9238
	P value	0.0029	0.0006		P value	0.0150	0.0000
Flower Biomass	F value	0.6292	3.2244	Aboveground Biomass	F value	1.0072	1.0753
	P value	0.6441	0.0201		P value	0.4131	0.3792
Branch Biomass	F value	1.1464	0.2590	Tuber Biomass	F value	6.2999	89.7111
	P value	0.3462	0.9027		P value	0.0004	0.0000

### Influence of root cutting time and radius on biomass in JA

**Figure 1:**
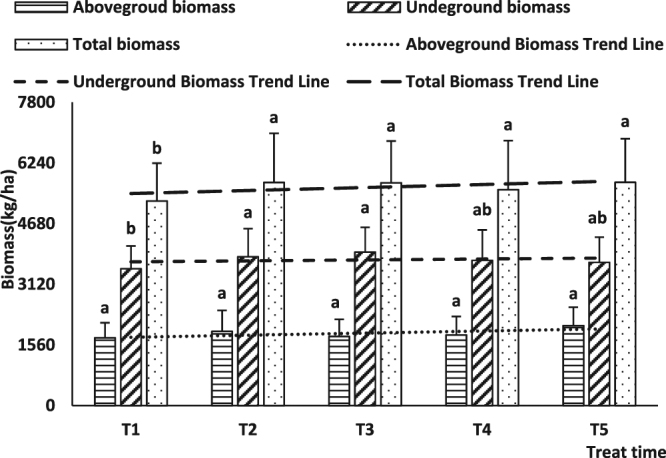
Influence of cutting time on biomass in JA.

**Figure 2:**
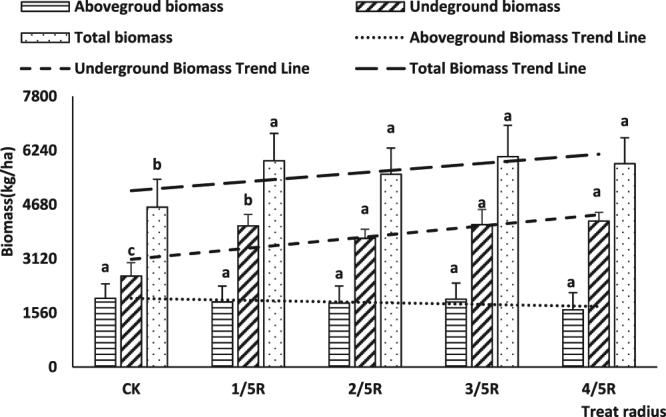
Influence of cutting radius on biomass in JA. Note: Lowercase letters indicate significant differences at the 0.05 level.

With a delay of cutting root time, the trend line of aboveground biomass, underground biomass, and total biomass changed gradually (Fig. 1). However, whereas the trend line of underground biomass and total biomass showed a gradually increasing trend, aboveground biomass showed a decreasing trend (Fig. 2).

Under different root cutting times, the value of total biomass of T2, T3, T4, and T5 was significantly higher than that of T1 (P < 0.05), and the value of underground biomass of T2 and T3 was also markedly higher than that of T1 (P < 0.05). In contrast, there were no differences among root cutting times on aboveground biomass. For different cutting radii, the total biomass for root cutting radii 1/5 R, 2/5 R, 3/5 R, and 4/5 R was markedly higher than in no cutting treatments (Control). Furthermore, there were no differences among different cutting radii for aboveground biomass, whereas the underground biomass of different root cutting treatments was significantly higher than that obtained with no cutting (Control), and the underground biomass obtained with root cutting radii of 2/5 R, 3/5 R, and 4/5 R was markedly higher than that obtained with 1/5 R.

### Influence if root cutting time and radius on organs biomass

#### Stem

When root cutting was delayed, stem biomass increased gradually. The values of T4 and T5 were markedly higher than those of other treatments (P < 0.05), and the value of T3 was significantly higher than that of T1 and T2 (P < 0.05). The stem biomass was improved by the root cutting treatment, and the stem biomass at cutting radii 1/5 R, 2/5 R, and 3/5 R was markedly higher than that of the control (P < 0.05), whereas the value at cutting radii 1/5 R and 2/5 R was higher than that of the control and at 4/5 R (P < 0.05) (Table 2).

**Table 2:** The influence of root cutting time and radius on organs biomass (kg/ha).

	stem biomass	leaf biomass	flower biomass	branch biomass	tuber biomass	root biomass
T1	391.46 ± 89.25c	490.54 ± 53.99a	248.02 ± 30.71a	614.37 ± 68.22b	2217.21 ± 292.40b	2620.04 ± 675.27b
T2	409.70 ± 82.03c	445.51 ± 72.43ab	260.24 ± 47.96a	796.80 ± 793.93ab	2450.17 ± 383.95a	2738.65 ± 916.34b
T3	509.48 ± 134.39b	295.48 ± 50.26c	252.42 ± 39.95a	289.34 ± 63.22ab	2410.16 ± 379.43a	2963.40 ± 855.29a
T4	584.14 ± 114.13a	366.56 ± 55.93bc	239.33 ± 60.64a	627.37 ± 83.64b	2350.94 ± 319.95a	2893.87 ± 810.99a
T5	603.92 ± 144.29a	353.67 ± 59.76bc	231.40 ± 46.53a	868.41 ± 138.46a	2395.48 ± 334.56a	2590.66 ± 782.00b
1/5 R	558.62 ± 133.12a	362.25 ± 102.29b	251.04 ± 28.15a	704.86 ± 72.38b	2417.72 ± 174.10b	3315.10 ± 643.80a
2/5 R	538.07 ± 128.91a	395.14 ± 133.94b	236.65 ± 74.95ab	676.34 ± 130.32b	2290.33 ± 143.10b	2846.91 ± 441.69b
3/5 R	522.38 ± 142.70ab	371.87 ± 136.11b	257.62 ± 46.83ab	803.65 ± 156.02ab	2472.90 ± 191.30b	3277.89 ± 655.78a
4/5 R	457.78 ± 125.10bc	292.60 ± 72.16b	208.95 ± 45.02b	693.03 ± 178.41b	2788.19 ± 188.44a	2847.42 ± 436.89b
Control	421.84 ± 82.58c	529.91 ± 91.06a	277.14 ± 79.90a	752.43 ± 67.95a	1854.81 ± 103.25c	1519.34 ± 206.07c

Note: Lowercase letters indicate a significant difference at the 0.05 level.

#### Leaf

Leaf biomass showed an initial decrease and then increased with the time of root cutting delay, and the lowest value was recorded at the 80 seedling stage (T3). Leaf biomass at the 50 (T1) and 65 (T2) seedling stages was markedly higher than that at the 80 (T3) seedling stage (P < 0.05), leaf biomass at the 50 (T1) seedling stage was higher than that at the 80 (T3), 95 (T4), and 115 (T5) seedling stages (P<0.05), and leaf biomass at the 65 (T2) seedling stage was higher than that at the 80 (T3) seedling stage (P < 0.05). The leaf biomass of all treatments was significantly decreased compared with the control (no cutting) (P < 0.05). Furthermore, leaf biomass showed an initial increase and then decreased from cutting radius 1/5 R to 4/5 R, but it did not show a difference among treatments (Table 2).

#### Flower

No differences among root cutting times were observed with respect to flower biomass. Flower biomass was decreased by different cutting radii, and the value at cutting radius 4/5 R was significantly lower than that obtained for the control (P < 0.05), whereas there were no differences among the other treatments (Table 2).

#### Branch

The largest branch biomass was obtained at the 115 (T5) seedling stage, which was significantly higher than that at T1 and T4 (P < 0.05), although it did not show a marked difference to that at T2 and T3. The branch biomass showed a gradual increase with an increase in cutting radius, with the largest value being obtained at a cutting root radius of 3/5 R, which was significantly higher than that obtained at 1/5 R, 2/5 R, and 4/5 R (P < 0.05). However, there were no significant differences between no cutting (Control) and root cutting treatments (Table 2).

#### Tuber

The tuber biomasses obtained at the 65 (T2), 80 (T3), 95 (T4), and 115 (T5) seedling stage were all significantly higher than that obtained at the 50 (T1) seedling stage (P < 0.05). Tuber biomass was significantly improved by different cutting radii (P < 0.05), and the value gradually improved from cutting radius 1/5 R to 4/5 R, although was not significantly different among cutting radii 1/5 R, 2/5 R, and 3/5 R (P < 0.05) (Table 2).

#### Root

The root biomass was significantly improved under conditions of different root cutting radius (P<0.01). and the values at cutting radii 1/5 R and 3/5 R were higher than those at 2/5 R and 4/5 R (P < 0.05), whereas there were no difference among other treatments. Root biomass showed an initial increase and then decreased with a delay in root cutting time, and the values obtained at T3 and T4 were significantly higher than those obtained with other treatments (P < 0.05). There was no difference between T3 and T4, or among T2, T3, and T5 (Table 2).

### Influence of root cutting radius and time on the root-shoot ratio in JA

**Figure 3:**
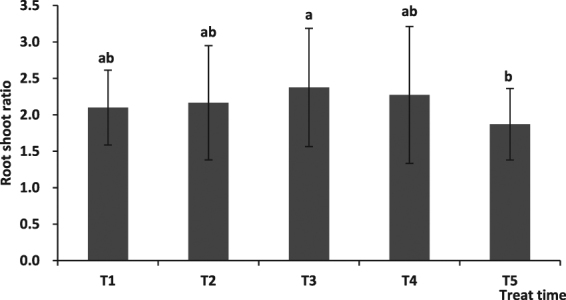
The effect of cutting root time on root/shoot ratio.

The root to shoot ratio showed an initial increase and then decreased with a delay of cutting root time. and the value at the highest value was obtained at the 80 seedling stage (T3), which was significantly higher than that at the 115 seedling stage (T5) (P < 0.05), although did not differ significantly from that recorded for the other treatments (Fig. 3).

**Figure 4:**
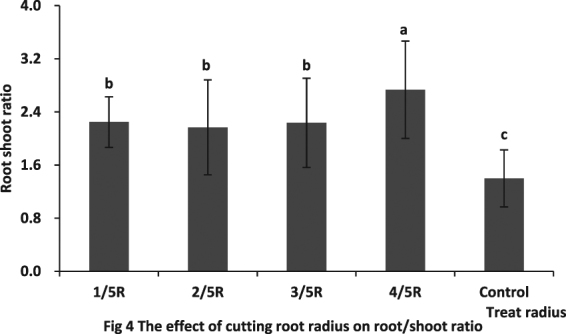
The effect of cutting root radius on root/shoot ratio. Note: Lowercase letters indicate a significant difference at the 0.05 level.

The root to shoot ratio was markedly improved by the different root cutting radii (P < 0.05). The highest value was obtained at root cutting radius 4/5, which was significantly higher than that recorded for root cutting radii 1/5, 4/5 and 3/5 (P < 0.05). There were no differences among root cutting radius 1/5, 2/5, and 3/5 treatments (Fig. 4).

### Influence of root cutting radius and time on the stem-leaf ratio in JA

**Figure 5:**
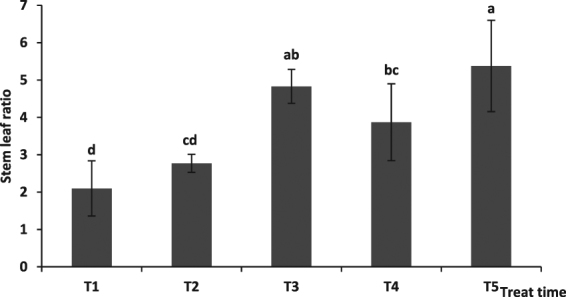
The effect of cutting root time on stem leaf ratio.

The stem to leaf ratio at the 80 (T3), 95 (T4), and 115 (T5) seedling stages was markedly higher than that at the 50 seedling stage (T1) (P < 0.05), that at the 115 seedling stage (T5) was higher than that at the 95 (T4) seedling stage, and that at the 85 (T3) and 95 (T4) seedling stages was higher than that at the 50 (T1) seedling stage (P < 0.05) (Fig. 5).

**Figure 6:**
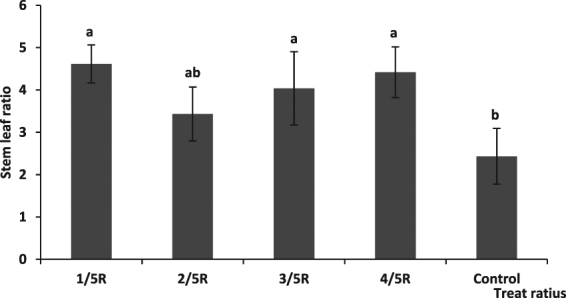
The effect of cutting root radius on stem leaf ratio. Note: Lowercase letters indicate a significant difference at the 0.05 level.

The stem to leaf ratio was improved by different root cutting radii, with the value at cutting radii 1/5 R and 4/5 R being markedly higher than that of the control (*P* < 0.05), and that at cutting radius 3/5 R being higher than that of the control (P < 0.05). There were no differences among the stem to leaf ratios recorded for cutting radii 1/5 R, 2/5 R, 3/5 R, and 4/5 R (Fig. 6).

### Correlation Analysis

There were significant positive correlation between many factors, including stem biomass and root biomass, stem biomass and total biomass, stem biomass and stem-leaf ratio, branch biomass and total biomass, tuber biomass and root biomass, tuber biomass and total biomass, root biomass and total biomass, root biomass and stem-leaf ratio, root biomass and shoot-root ratio, and stem-leaf ratio and shoot-root ratio (P < 0.01). Furthermore, there were significant difference between leaf biomass and flower biomass, flower biomass and branch biomass, tuber biomass and stem-leaf ratio, and total biomass and stem-leaf ratio (P < 0.05). There were also significant negative correlation between many factors, including stem biomass and leaf biomass, leaf biomass and tuber biomass, leaf biomass and root biomass, leaf biomass and stem-leaf ratio, leaf biomass and shoot-root ratio, flower biomass and shoot-root ratio, and branch biomass and shoot-root ratio (P < 0.01). There were also significant negative difference between flower biomass and tuber biomass and flower biomass and stem-leaf ratio (P < 0.05) (Table 3).

**Table 3:** Correlation Analysis of different organs biomass.

	Stem	Leaf	Flower	Branch	Tuber	Root	Total Biomass	Stem-leaf ratio	Shoot-root ratio
Stem	1								
Leaf	−0.318**	1							
Flower	−0.046	0.289*	1						
Branch	0.014	0.224	0.265*	1					
Tuber	0.125	−0.455**	−0.245*	−0.005	1				
Root	0.344**	−0.360**	−0.153	−0.031	0.590**	1			
Total Biomass	0.373**	−0.189	−0.003	0.372**	0.667**	0.882**	1		
Stem-leaf ratio	0.700**	−0.746**	−0.261*	−0.146	0.288*	0.352**	0.255*	1	
Shoot-root ratio	−0.063	−0.547**	−0.483**	−0.669**	0.612**	0.496**	0.198	0.300**	1

**Indicates a significant difference at the 0.01 level.

*Indicates a significant difference at the 0.05 level.

## Discussion

In this study, a portion of the root system was removed more or less through root cutting, which resulted in some variation in tuber yield, organ biomasses, stem-leaf ratio, and root-shoot ratio. The experimental results showed that root cutting could effectively decrease the biomass of leaves, flowers, and branches. The reason for these responses is probably that JA could not take up adequate water and nutrients for the growth of aboveground organs due to a decrease in existing root surface areas caused by root cutting. It is also likely that root cutting caused an imbalance in the root to shoot ratio; however, it is unlikely that plant can support rapid new root development and maintain consistent shoot growth at the same time, but initially expend more resources on root growth rather than on shoot growth^15,16^.

The tuber is the main organ of JA, which is also the main source of bioethanol and inulin^3^. Tuber biomass was improved by root cutting and the value increased with an increase in the delay of root cutting. This response can probably be attributed to the growth process of JA, which includes vegetative growth and reproductive growth stages^4^. In the pre-growth stage (vegetative growth stage), the vegetative organs, including stems, leaves, and roots, show rapid growth, whereas in the reproductive growth stage the reproductive organs (tubers and flowers) show rapid growth. Accordingly, with early root cutting, when tubers and flowers had not appeared, there was a greater influence on stems, leaves, and root. With later root cutting, JA had entered the reproductive growth stages, when tubers and flowers had appeared and were growing rapidly, and so root pruning can promote tuber accumulation during reproductive growth. Therefore, tuber biomass was highest with the 115 seedling stage treatment, whereas it was lowest with the 50 seedling stage treatment. The change in leaf biomass showed the opposite trend: the value was lowest with the 115 seedling stage treatment and highest with the 50 seedling stage treatment.

Compared with the control, the root to shoot ratio was higher under the different root cutting radius treatments. We can’t see the reason from the effect of cutting root on the biomass of JA, but cutting root improved effectively the biomass of underground organs such as root and tuber or decreased the biomass of leaf and flower. Thus, the root to shoot ratio of JA under different root cutting radius treatments was higher than that of control. The response could be attributed to the fact that cutting roots stimulates the growth of underground organs, accelerates the transport of photosynthate from leaves to underground parts, and decreases the leaf biomass. The effect of different root cutting times on the root to shoot ratio of JA initially decreases and then increase. The reason for this pattern is that the seedling stages 50, 65, and 80 are in the vegetative growth stage, during which effective nutrient transport to underground parts is stimulated and the emergence of new roots is promoted. Conversely, at seedling stages 95 and 115, plants are in the reproductive growth stages, during which tubers and flowers form and vegetative growth is at a standstill.
